# Virus-Induced CD8^+^ T-Cell Immunity and Its Exploitation to Contain the SARS-CoV-2 Pandemic

**DOI:** 10.3390/vaccines9080922

**Published:** 2021-08-18

**Authors:** Maurizio Federico

**Affiliations:** National Center for Global Health, Istituto Superiore di Sanità, 00161 Rome, Italy; maurizio.federico@iss.it

**Keywords:** cross-presentation, CD8^+^ T-cell immunity, SARS-CoV-2, extracellular vesicles

## Abstract

The current battle against Severe Acute Respiratory Syndrome (SARS)-Coronavirus-2 benefits from the worldwide distribution of different vaccine formulations. All anti-SARS-CoV-2 vaccines in use are conceived to induce anti-Spike neutralizing antibodies. However, this strategy still has unresolved issues, the most relevant of which are: (i) the resistance to neutralizing antibodies of emerging SARS-CoV-2 variants and (ii) the waning of neutralizing antibodies. On the other hand, both pre-clinical evidence and clinical evidence support the idea that the immunity sustained by antigen-specific CD8^+^ T lymphocytes can complement and also surrogate the antiviral humoral immunity. As a distinctive feature, anti-SARS-CoV-2 CD8^+^ T-driven immunity maintains its efficacy even in the presence of viral protein mutations. In addition, on the basis of data obtained in survivors of the SARS-CoV epidemic, this immunity is expected to last for several years. In this review, both the mechanisms and role of CD8^+^ T-cell immunity in viral infections, particularly those induced by SARS-CoV and SARS-CoV-2, are analyzed. Moreover, a CD8^+^ T-cell-based vaccine platform relying on in vivo engineered extracellular vesicles is described. When applied to SARS-CoV-2, this strategy was proven to induce a strong immunogenicity, holding great promise for its translation into the clinic.

## 1. Introduction

The immune system can react against virus attack essentially through three lines of defense, i.e., innate immunity (interferons, natural killer cells), humoral adaptive immunity (antibodies, memory B lymphocytes), and cellular adaptive immunity (CD8^+^ T lymphocytes). The optimal efficiency of each immunity branch can be, per se, sufficient to counteract the threat from virus infections. To date, the antiviral potentialities of viral antigen-specific CD8^+^ T lymphocytes have been less considered in terms of both prophylactic and therapeutic antiviral interventions.

Typically, the CD8^+^ T-cell immune response begins to mount due to the degradation of cell-expressed proteins and the exposition of the produced peptides on the major histocompatibility complex (MHC) Class I of professional antigen-presenting cells (APCs), most often dendritic cells (DCs). While this process accounts for the CD8^+^ T-cell immunity induced against viruses infecting and expressing into professional antigen-presenting cells (APCs), it cannot explain the CD8^+^ T-cell immunity elicited against viruses unable to express into these cells. This conundrum was solved by the identification and characterization of cross-presentation as the mechanism addressing exogenous antigens to degradation and association with MHC Class I molecules, ultimately leading to CD8^+^ T lymphocyte cross-priming [1]. In several instances, cross-presentation is supposed to be on the basis of the induction of the antiviral CD8^+^ T-cell immune response.

In this review, the molecular mechanisms underlying the cross-presentation process are synthetically depicted. In addition, the role of CD8^+^ T-cell immunity in the pathogenesis induced by diverse viral infections, particularly those induced by Severe Acute Respiratory Syndrome (SARS)-Coronavirus (CoV) and SARS-CoV-2, is analyzed. Diseases induced by these viruses in many instances are marked by severe lung inflammation, which can influence the functions of CD8^+^ T resident memory (rm) cells supposedly generated by previous exposition to cognate viruses [2]. CD8^+^ Trm cells originate mainly from circulating effector memory CD8^+^ T cells and differentiate in tissues without returning to circulation [3]. CD8^+^ Trm cells are a frontline cell population in the immune response against respiratory viruses [4] by virtue of manifold functions, including direct antigen recognition, the release of inflammatory factors, and the recruitment of circulating memory CD8^+^ T cells [5]. The expected effects of SARS-CoV-2-induced inflammation on CD8^+^ Trm cell functions are here discussed.

Finally, both the mechanism and possible applications against Coronavirus Infectious Disease (COVID)-19 of an original vaccine platform based on antigen-specific CD8^+^ T-cell immunity induced by in vivo engineered extracellular vesicles are discussed.

## 2. Mechanisms of Cross-Presentation

**Figure 1:**
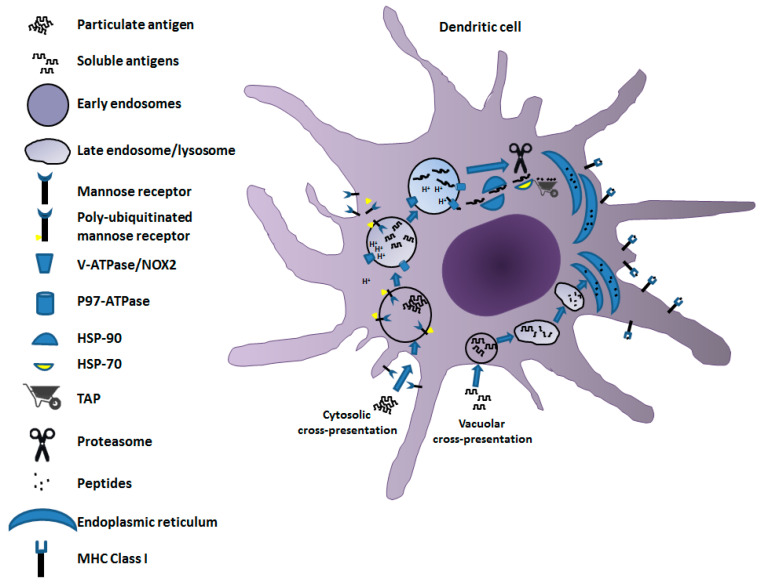
Mechanisms of cross-presentation. Both vacuolar (bottom flow) and cytosolic (upper flow) pathways are depicted. In the vacuolar cross-presentation pathway, after internalization by endo/pinocytosis, the antigen remains in intracellular compartments. It is degraded by the activity of cathepsin S, and the resulting peptides are translocated to ER to be loaded on MHC Class I molecules. In the cytosolic pathway, the antigen, more often in a particulate form, is internalized by endocytosis, thereby undergoing dissociation/denaturation in a mildly acidic pH regulated by the v-ATPase/NOX2 interaction. Denatured antigens are then transferred to cytosol through channels formed by p97 ATPase recruited to endosomes upon poly-ubiquitination of MR. Exported antigens bind chaperone Hsp90, and then are forwarded to proteasome degradation after interaction with Hsp70. Finally, peptides are translocated by TAP into ER, where they associate with the MHC Class I complex.

Antigen cross-presentation in DCs, i.e., the most active cell type in terms of cross-priming, is governed by two mechanisms: the vacuolar and the cytosolic pathways (Figure 1). The vacuolar cross-presentation pathway was originally described for bacterial antigens [6]. In this case, the products of antigen degradation to be associated with MHC Class I molecules are generated through a pathway developing entirely apart from cytosol. This conclusion was supported by the experimental evidence that MHC Class I cross-presentation of bacterial products resists the brefeldin A (BFA) treatment, which blocks the export of molecules from endosomes, and is independent of the activity of cytosolic effectors such as proteasome and transporter associated with antigen processing (TAP) [7], the latter delivering peptides from cytosol to endoplasmic reticulum (ER) for MHC Class I association. Once internalized, the antigen remains confined in intracellular compartments, degraded by the activity of cathepsin S, and loaded on MHC Class I molecules [8].

On the other hand, the cytosolic pathway was first demonstrated for particulate antigens [9]. It depends on TAP activity and is sensitive to BFA treatment. The cytosolic export of the antigen from the endocytic compartment is the distinctive signature of this pathway. Particulate antigens are supposed to be dissociated by a mildly acidic pH into endo-phagosomes before translocation to the cytosol. To this aim, limited activity of membrane-associated vacuolar(v)-ATPase proton pump, regulated by the interaction with the Rab27a-dependent NOX2 activation, leads to a fine tuning of endosomal pH [10,11]. This is necessary to avoid antigen degradation that is too extensive due to an excessively low pH. The transfer of antigen to cytosol is regulated by the activity of the AAA (ATPase associated with diverse cellular activities) p97 [12,13,14], also known as valosin-containing protein (VCP). It is recruited to endosomes upon poly-ubiquitination of the mannose receptor (MR) occurring through the attachment of a poly-ubiquitin chain to lysine 48 of its cytosolic region upon cell internalization from the plasma membrane [15]. P97 ATPase generates endosome to cytosol channels by forming hexameric rings throughout the endosomal membrane, meanwhile providing the energy required for the delivery of an unfolded antigen from the endosomal lumen to the cytosol. Exported antigens are protected from premature degradation through binding with chaperone heat-shock protein (Hsp)90 [16,17]. Therefore, the antigen is forwarded to proteasome degradation after interaction with Hsp70 [18].

Whatever the means by which peptides are produced, once delivered to ER they are associated and presented on the MHC Class I complex following the mechanisms common to endogenous antigens.

## 3. The Impact of CD8^+^ T-Cell Immunity in Viral Diseases

The majority of literature data demonstrate that the virus-specific CD8^+^ T-cell immunity plays a pivotal role in the protection from several viral diseases. For instance, the infusion of CD8^+^ T cytotoxic lymphocytes (CTLs) specific for six proteins of Ebola virus was shown to protect mice from lethal virus challenge [19], and a similar result was obtained with CTLs specific for nucleoprotein (NP) protein only [20]. In humans, the analysis on survivors of the recent Ebola virus epidemic (2013–2016) showed that basically all subjects retained strong CD8^+^ T-cell immunity against several viral antigens. In particular, 96% of subjects developed CD8^+^ T-cell responses to the viral NP, 77% to VP24, 69% to VP40, 42% to VP35, and 38% to GP [21].

Several studies also highlighted the importance of CD8^+^ T cells in protecting against infection by West Nile virus (WNV). In particular, mice deficient in either the production of CD8^+^ T lymphocytes or MHC class I expression exhibited much greater mortality than wild-type mice upon WNV infection [22]. The infecting virus replicated to a greater extent in brains of knockout mice, and persisted in surviving mice for more than 30 days post-infection. Consistently, the ability of CD8^+^ T cells to directly kill virally infected cells was shown to be a vital element in the control of WNV, as demonstrated by the evidence that mice lacking either perforin, Fas-L, or TRAIL expression exhibited increased mortality following infection [23].

The relevance of the virus-specific CD8^+^ T-cell immunity in the protection from different subtypes of Influenza A virus (IAV) was originally demonstrated in a study including 63 volunteers inoculated with live IAV [24]. More recently, it was shown that memory CD8^+^ T lymphocytes specific for antigenic peptides derived from structural viral proteins (i.e., M1, NP) conferred protection from infection of both homologous and heterologous viral strains [25]. Notably, the presence of a population of virus-specific, lung CD8^+^ Trm cells was found necessary for the optimal heterosubtypic immunity [26].

Besides protecting from the attack of subtypes of the same virus species, the CD8^+^ T-cell immunity also has the potential to counteract the spread of viruses of different, yet antigen-related, species. For instance, adoptive transfer and cell depletion studies demonstrated that Dengue virus-specific CD8^+^ T cells mediated cross-protection against Zika virus. Conversely, passive transfer analysis indicated that the immune sera did not possess such a cross-protective effect [27].

In conclusion, literature data consistently support the idea that the CD8^+^ T-cell immunity branch plays a key role in the natural defense against virus attack, meanwhile showing cross-protection activity.

## 4. The CD8^+^ T-Cell Immune Response in SARS-CoV and SARS-CoV-2 Infections

An emerging coronavirus, identified as SARS-CoV, was the causative agent of the outbreak in 2002/2003, which was extinguished within 6–7 months. A related coronavirus, the Middle East Respiratory Syndrome (MERS)-CoV, emerged in 2012 in Middle East countries and is still endemic. Now, the world is facing an unprecedented pandemic caused by a novel coronavirus, the SARS-CoV-2. The strict genetic and pathogenetic similarities with SARS-CoV have been of much help in deciphering many aspects of the biology of SARS-CoV-2 and correlated immune responses.

The case fatality rate for SARS-CoV infections was about 10–15% for <60-year-old patients, and as high as more than 50% for older patients [28]. In seminal studies, Perlman’s group demonstrated that both CD4^+^ and CD8^+^ T-cell immunities were necessary and sufficient to block the infection in mice by a mouse-adapted SARS-CoV strain in the absence of antibody response [29]. More stringently, virus-specific CD8^+^ T memory cells were proven to be sufficient to protect mice from infection [30]. In humans, the acute phase of SARS-CoV disease associated with >80% reduction in both CD4^+^ and CD8^+^ T lymphocytes as well as defects in their activation, ultimately leading to a delayed adaptive immune response against the virus [31,32]. Such massive lymphocyte depletion is a consequence of immunologic mechanisms (e.g., elevated cytokine production, inhibition of APC functions) [33,34], rather than a direct cytopathic effect on lymphocytes. Concerning the antiviral immune adaptive responses, virus-specific neutralizing antibodies and memory B-cells have been shown to become undetectable within 2 years after symptom onset [35,36]. On the contrary, the presence of NP-specific CD8^+^ T lymphocytes within peripheral blood mononuclear cells (PBMCs) of survivors was demonstrated to persist for as long as 17 years after recovery [37].

In summary, both experimental evidence and clinical evidence highlight the long-lasting protective role that the CD8^+^ T-cell immunity plays in the natural response to SARS-CoV infection.

The results from a number of studies on animals also well depicted the role that CD8^+^ T-cell immunity plays in the recovery from SARS-CoV-2 infection. For instance, mice that became susceptible to virus infection through a previous intranasal treatment with an ACE2-expressing adenoviral vector controlled the viral spread by virtue of the activation of virus-specific CD4^+^ and CD8^+^ T cells in the absence of neutralizing antibodies [38]. Importantly, in this study, the T-cell immunity raised against SARS-CoV-2 was shown to also be effective against the related SARS-CoV. Data from additional investigations showed that rhesus macaques treated with a peptide-based vaccine targeting MHC Class I epitopes of the SARS-CoV-2 NP protein failed to show symptoms after viral infection in the presence of a reduced viral load in both nasal swabs and bronchoalveolar fluids [39]. Furthermore, a seminal study on rhesus macaques demonstrated that the depletion of CD8^+^ T cells after a first virus challenge nullified the protective effect of natural immunity against a virus re-challenge carried out after the waning of neutralizing antibodies [40]. The authors concluded that the antiviral CD8^+^ T-cell immunity can control the virus spread in the context of suboptimal levels of neutralizing antibodies.

In humans, SARS-CoV-2-specific CD8^+^ T cells readily develop after infection, and were most frequently found in patients presenting mild symptoms. Upon infection, most frequent virus-specific CD8^+^ T cells are generated against NP, ORF1ab, ORF3a, S, and M viral proteins [41]. CD8^+^ T-cell epitopes most commonly identified in convalescent patients, and classified according to MHC Class I alleles, are reported in Table 1.

Interestingly, virus-specific memory CD8^+^ T cells exhibited functional characteristics as strong as those of influenza virus-specific CD8^+^ T cells. SARS-CoV-2-specific memory CD8^+^ T cells were also detectable in convalescent individuals that did not develop anti-SARS-CoV-2 antibodies [42], a result strictly reminiscent of what was previously observed in SARS-CoV survivors [29]. Concerning the role of CD8^+^ T-cell immunity in the recovery from SARS-CoV-2 infection, data from humans appeared to be consistent with the evidence obtained in animal models. Generally speaking, the presence of virus-specific CD8^+^ T lymphocytes is associated with a better recovery from the disease [43,44], whereas, on the contrary, the levels of antibody response parallels the disease severity [45]. Consistently, Tan and coll. reported that the prompt activation of SARS-CoV-2-specific CD8^+^ T lymphocytes, particularly those specific to the NP protein, was associated with disease control [46]. In another study, patients with severe disease showed a drastic reduction in total CD8^+^ T-cell counts with no T-cell immunity against S and NP, which, conversely, were present in patients recognizing mild diseases [47]. Interestingly, low levels of virus-specific CD8^+^ T-cell immune response have been found in infected subjects over the age of 80 years, i.e., a class of patients frequently developing a severe disease [47].

An additional piece of evidence concerning the relevance of CD8^+^ T-related immunity comes from studies performed on infected patients bearing hematologic cancers [48]. Oncologic subjects having significant impairment of B cells, but preserving CD8^+^ T-cell counts, showed lower viral loads and reduced mortality upon SARS-CoV-2 infection compared to what was observed in homologous patients with low CD8^+^ T-cell counts. In addition, the depletion of B cells in patients with hematologic cancers did not associate with increased COVID-19-related mortality [48]. These clinical observations further enforced the idea that CD8^+^ T cells can compensate for deficient humoral immunity, thus being beneficial for COVID-19 recovery.

Of major relevance, and different to what was observed with the antibody response, the SARS-CoV-2-specific CD8^+^ T-cell immunity has been proven to maintain its efficacy in the presence of the amino acid substitutions occurring in emerging viral variants. As a consequence, the CD8^+^ T-cell immunity induced by a strain is expected to be active against viral variants as well. This conclusion was drawn from the study of Redd and coll., who examined 30 convalescent patients and found that only one out of 45 mutations in the B.1.351 variant (beta-variant of concern, VOC) overlapped with a low-prevalence CD8^+^ T epitope [49]. Accordingly, Tarke and coll. demonstrated that the striking majority of SARS-CoV-2 CD8^+^ T-cell epitopes in COVID-19 convalescent subjects is not affected by the mutations found in B.1.1.7 (alpha-VOC), B.1.351, P.1 (gamma-VOC) and B 1.427/B.1.429 (epsilon-variant of interest, VOI) viral strains [50]. Furthermore, a study carried out on most prevalent HLA types identified shared epitopes that were found located in conserved regions, with only 3 out of these 29 epitopes being located in S protein.

In other words, which is the protein undergoing mutation most frequently [51]. Finally, using an innovative αβ T-cell staining platform, Mallajosyula and coll. demonstrated the presence of higher levels of CD8^+^ T cells specific for conserved coronavirus epitopes in patients showing mild symptoms than in those with severe disease [2].

In several cases, severe lung inflammation is associated with COVID-19 disease. Inflammation extents can influence the functions of CD8^+^ Trm lymphocytes [54]. In particular, low inflammation levels hinder both cell survival and the input of effector T lymphocytes from circulating cells, whereas a strong inflammation state leads to a diversion towards the effector terminal phenotype and a reduction in the transition from effector to memory cells. Conversely, intermediate levels of inflammation coupled with adequate levels of type I signals would favor the memory functions of CD8^+^ Trm, including their potential to be reactivated. On this basis, a SARS-CoV-2-induced strong inflammation is expected to heavily impair the activity of CD8^+^ Trm generated by previous exposition to cognate viruses [2], thus inhibiting an effective protection against the virus attack. Therefore, it is conceivable that, in some instances, a pharmacologic control of inflammation at early times after symptom onset would contribute to a mild evolution of the disease by favoring the antiviral functions of virus-specific CD8^+^ Trm.

Taken together, these arguments demonstrate the key role of CD8^+^ T-cell immunity in the COVID-19 recovery. Of paramount relevance, the efficiency of this immunity is not impaired by mutations accumulating in circulating viral variants.

## 5. A Candidate CD8^+^ T-Cell-Based Vaccine to Fight COVID-19

Although a strong CD8^+^ T-cell response should be part of any vaccine against SARS-CoV-2, no reliable technology for the induction of CD8^+^ T-cell immunity has been validated for humans to date. The use of adenoviral vector-based vaccine platforms generated promising results in several preclinical settings. Unfortunately enough, however, the results from clinical trials with anti-SARS-CoV-2 vaccines produced through this technology did not meet the anticipated outcomes in terms of the induction of CD8^+^ T-cell immunity [55,56].

Attempting to fill this gap, we developed an original strategy that would be exploited to fight SARS-CoV-2 spread. It is based on the induction of antigen-specific CTL immunity by means of in vivo engineered extracellular vesicles (EVs).

All cell types constitutively release nanovesicles, which are key players in intercellular communication [57]. Collectively, they are referred to as EVs. When produced by healthy cells, EVs can be distinguished in exosomes and microvesicles. Exosomes are lipid bilayered vesicles of 50–200 nanometers in diameter and form intracellularly upon inward invagination of endosome membranes. Intraluminal vesicles (ILVs) produced in this way form multivesicular bodies (MVBs) that can traffic either to lysosomes for degradation, or to the plasma membrane. In the latter case, MVBs release nanovesicles in the extracellular milieu after fusion with the plasma membrane. Microvesicles are up to 1000 nanometers in diameter and possess both physical and biochemical features similar to those of exosomes; however, they are generated through direct extrusion of the plasma membrane. EVs can spontaneously upload DNA, messenger RNA, non-coding RNA, microRNAs, and proteins that can be functional once delivered into target cells. Hence, EVs can be considered multi-molecular messengers acting in both autocrine and paracrine ways [58].

We developed a vaccine platform based on the intramuscular injection of a DNA vector coding for a biologically inactive Human Immunodeficiency Virus (HIV)-Type 1 Nef protein (Nef^mut^) with an unusually high efficiency of incorporation into EVs even when foreign polypeptides are fused to its C-terminus [59]. Both N-terminal myristoylation and palmitoylation fasten Nef^mut^ to the luminal membrane leaflets and are critical for its abundant uploading in EVs [60]. Nanovesicles containing Nef^mut^-fused antigens released by muscle cells can freely circulate into the body and be internalized by APCs. EV-associated antigens are then cross-presented to prime antigen-specific CD8^+^ T cells [61]. Notably, a Nef^mut^ isotype with a significant C-terminal truncation maintains both EV-anchoring and immunogenic properties, thus representing a safer alternative for use in clinic [62].

Even if both cross-presentation and cross-priming following the entry of Nef^mut^ engineered EVs have been demonstrated in in vitro/ex vivo experiments [63,64], the exact events, from the attachment on the APCs of engineered EVs to antigen cross-presentation, still have to be clarified in full. Glycomic studies demonstrated that the surface of EVs, particularly exosomes, contains high amounts of mannose and other classes of N-linked oligosaccharides [65,66], making them suitable ligands for the mannose receptor (MR). This receptor is expressed on the surface of immature dendritic cells (DCs), liver sinusoidal endothelial cells, M2 macrophages, and other tissue macrophages. The key role of mannose/MR interplay in EV entry into DCs was highlighted by the observation that mannose-enriched EVs displayed quite elevated uptake by murine DCs [67].

MR is a 175 kilodalton type I integral membrane protein belonging to the family of C-type lectin receptors. It binds glyco-conjugates terminated in mannose, fucose, or *N*-acetil-β-d-glucosamine in a calcium-dependent manner [68]. MR is a highly effective clathrin-dependent endocytic receptor that constantly recycles between the plasma membrane and early endosomal compartment [69]. Most of an MR is intracellular, while only ∼15% of the cellular pool can be found on the cell surface. Like other members of the C-type lectin receptor family, MR undergoes conformational changes upon ligand binding or as pH decreases in intracellular compartments [70]. Once acidification takes place in the endosomal compartment, MR dissociates from its ligands, thereby recycling back to the plasma membrane.

**Figure 2:**
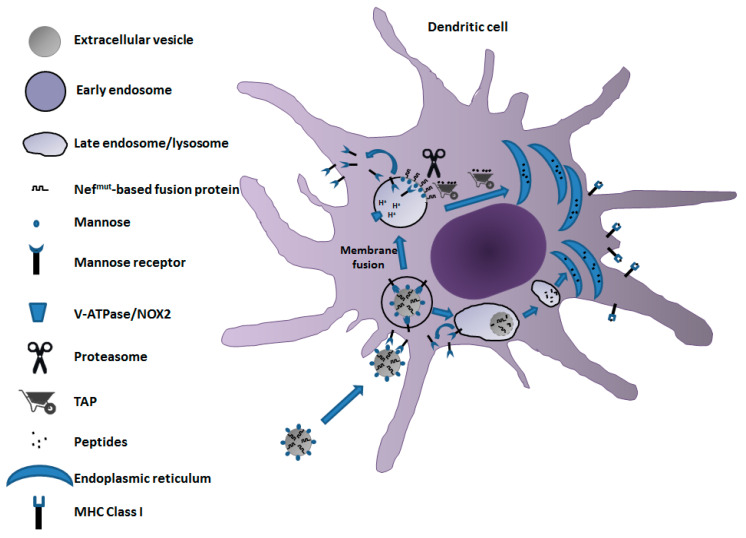
A model for the mechanism of cross-presentation of Nef^mut^-based antigens delivered by engineered EVs. EVs expressing mannose on their membrane and incorporating high levels of Nef^mut^-based fusion products bind to MR expressed by DCs. EV–DC interaction results in EV cell internalization by endocytosis. The endosome acidification leads to conformation change of the mannose receptor and its recycling toward the cell membrane. Cross-presentation of peptides from Nef^mut^-based fusion products can result from the vacuolar (bottom flow) and/or cytoplasmic (upper flow) pathways. In the latter case, endosome and EV membranes undergo fusion, ultimately leading to a single-membrane endosomal body with membrane-associated Nef^mut^-based fusion products exposed to cytoplasm. In this way, Nef^mut^-based fusion proteins become available for proteasome-mediated degradation. The resulting peptides are transported by TAP into ER, where they associate with the MHC class I complex to initiate the cross-priming process.

On the basis of these consolidated data of literature, a model for the mechanism of the cross-presentation of antigens uploaded in Nef^mut^-engineered EVs can be envisioned (Figure 2). After release from muscle cells, Nef^mut^-engineered EVs, by virtue of their expected high mannose content in membrane, bind an MR of immature DCs. Subsequent EV cell internalization can be followed by degradation into late endosomes/lysosomes, ultimately leading to the delivery of peptides to ER for complexing with MHC Class I at the completion of the vacuolar cross-presentation pathway. Alternatively, the endosome-internalized EV can undergo fusion with membranes of endosomes, similar to what was described for several viruses [71,72]. In this way, Nef^mut^-based fusion products are exposed to cytoplasm, hence becoming vulnerable to the proteasome degradative activity. Resulting peptides can be then translocated to ER by TAP for their complexing with MHC Class I molecules to initiate cross-priming events. Although this mechanism may be conceivable considering the N-terminal binding with the EV membrane of Nef^mut^-related products, experimental confirmations are still needed.

Nef^mut^-based strategy has already been demonstrated to be effective against both transplantable [73] and ectopic [64] tumors in view of a strong induction of tumor-specific CTL activity. To apply this technology to a design of anti-SARS-CoV-2 vaccine, DNA vectors expressing the products of fusion between Nef^mut^ and different viral antigens, namely, N- and C-terminal moieties of S (referred to as S1 and S2), M, and N, were generated. When the DNA vectors were injected in mice either alone or in combination, a strong antigen-specific CD8^+^ T-cell immunity became detectable in spleens and, most importantly, in lung airways [74].

For obvious reasons related to COVID-19 pathogenesis, the detection of valuable levels of virus-specific CD8^+^ T cells in lungs was of striking relevance. Generally speaking, resident CD8^+^ T cells in lungs are maintained independently of the pool of circulating CD8^+^ T cells through homeostatic proliferation aimed at replenishing the continuous loss of cells through intraepithelial migration towards lung airways [75]. On this basis, one can hypothesize that in the Nef^mut^-system the virus-specific CD8^+^ T cells can be generated upon cross-priming occurring at local, e.g., mediastinal, lymph nodes, assuming that freely circulating immunogenic EVs are captured by immature DCs of peripheral tissues. Upon EV internalization and cell activation, tissue-resident DCs would migrate to local lymph nodes, thereby switching the processes leading to CD8^+^ T-cell cross-priming. Consistent with what was observed in lungs, one may conceive that additional tissues relevant to SARS-CoV-2 pathogenesis, e.g., the gut and brain, would benefit from the EV-induced CTL immunity generated in distal lymph nodes.

## 6. Conclusions

The SARS-CoV-2 pandemic has given rise to the urgent need for vaccines and therapeutic interventions. Currently distributed vaccines have been conceived to induce neutralizing antibodies using the S viral protein as immunogen. However, the immunological correlates of protection against the viral infection still remain unknown. Most likely, a coordinated action of CD4^+^ T cells, CD8^+^ T cells, and neutralizing antibodies is necessary to control SARS-CoV-2 infection. Current vaccination strategies exclusively rely on anti-S neutralizing antibodies, which, however, wane in a few months. In addition, they showed greatly reduced efficacy against some emerging VOCs. To fill this gap, durability and cross-reactivity would be distinctive features that must be offered by next-generation anti-SARS-CoV-2 vaccines.

## Figures and Tables

**Table 1:** Most common SARS-CoV-2 CD8^+^ T-cell epitopes in humans^a^.

MHC Class I Allele	Viral Protein	Epitope Sequence	% of Positivity and [Reference]
A*01:01	ORF1ab	PTDNYITTY	60% [51]
A*01:01	ORF1ab	TTDPSFLGRY	83% [45]; 80% [51]
A*01:01	ORF1ab	NTCDGTTFTY	60% [51]
A*01:01	ORF1ab	CTDDNALAYY	100% [51]
A*02:01	ORF1ab	KLWAQCVQL	88.9% [51]
A*02:01	ORF1ab	ALWEIQQVV	88.9% [51]
A*11:01	ORF1ab	VTDTPKGPK	60% [51]
A*24:02	ORF1ab	VYIGDPAQL	70% [45]
B*07:02	ORF1ab	RPDTRYVL	80% [51]
B*40:01	ORF1ab	IEYPIIGDEL	58% [45]
A*01:01	S	LTDEMIAQY	50% [45]
A*02:01	S	YLQPRTFLL	77.8% [51]
A*03:01	S	KCYGVSPTK	100% [51]
A*24:02	S	QYIKWPWYI	60% [45]; 60% [51]
A*01:01	ORF3a	FTSDYYQLY	100% [51]
A*02:01	ORF3a	LLYDANYFL	88.9% [51]
A*02:01	ORF3a	ALSKGVHFV	55% [45]
A*24:02	ORF3a	VYFLQSINF	70% [45]; 80% [51]
A*01:01	M	ATSRTLSYY	60% [51]
A*11:01	M	ATSRTLSYYK	60% [51]
B*40:01	M	SELVIGAVIL	50% [45]
C*07:01	M	NRFLYIIKL	50% [45]
A*01:01	NP	FTSDYYQLY	100% [52]
A*01:01	NP	PTDNYITTY	70% [52]
A*01:01	NP	HTTDPSFLGRY	100% [52]
A*02:01	NP	YLQPRTFLL	65% [52]
A*03:01	NP	KTFPPTEPK	64% [45]; 100% [51]; 75% [52]
A*11:01	NP	KTFPPTEPK	100% [51]
A*11:01	NP	ATEGALNTPK	82% [45]
A*24:02	NP	NYNYLYRLF	65% [52]
A*24:02	NP	QYIKWPWYI	75% [52]
B*07:02	NP	RARSVSPKL	75% [52]
B*07:02	NP	SPRWYFYYL	80% [51]; 100% [52]
B*40:01	NP	MEVTPSGTWL	75% [45]

^a^ Epitopes detected in at least 50% of screened convalescent patients are reported. At least one among HLA-A*01:01, -A*02:01, -A*03:01, -A*11:01, -A*24:02, and -B*07:02 alleles is present in ~85% of the world population [53]. ^b^ ORF: open reading frame; S: Spike; M. Membrane; NP: Nucleoprotein.
